# How Early Is Early Multiple Sclerosis?

**DOI:** 10.3390/jcm13010214

**Published:** 2023-12-29

**Authors:** Sotiria Stavropoulou De Lorenzo, Christos Bakirtzis, Natalia Konstantinidou, Evangelia Kesidou, Dimitrios Parissis, Maria Eleptheria Evangelopoulos, Dina Elsayed, Eman Hamdy, Sameh Said, Nikolaos Grigoriadis

**Affiliations:** 1 Multiple Sclerosis Center, Second Department of Neurology, School of Medicine, Aristotle University of Thessaloniki, 54621 Thessaloniki, Greece; iradel7714@gmail.com (S.S.D.L.); nataliak95@gmail.com (N.K.); kesidoue@auth.gr (E.K.); dparisis@auth.gr (D.P.); ngrigoriadis@auth.gr (N.G.); 2 First Department of Neurology, Aeginition Hospital, National and Kapodistrian University of Athens, 11528 Athens, Greece; evangelopme@med.uoa.gr; 3 Department of Neuropsychiatry, Faculty of Medicine, Alexandria University, Alexandria 21311, Egypt; dr.dina237@gmail.com (D.E.); eman.ameen@alexmed.edu.eg (E.H.); drsamsaids@gmail.com (S.S.)

**Keywords:** multiple sclerosis, early treatment, RIS, multiple sclerosis prodrome, disease progression, laboratory biomarkers, neuroimaging biomarkers

## Abstract

The development and further optimization of the diagnostic criteria for multiple sclerosis (MS) emphasize the establishment of an early and accurate diagnosis. So far, numerous studies have revealed the significance of early treatment administration for MS and its association with slower disease progression and better late outcomes of the disease with regards to disability accumulation. However, according to current research results, both neuroinflammatory and neurodegenerative processes may exist prior to symptom initiation. Despite the fact that a significant proportion of individuals with radiologically isolated syndrome (RIS) progress to MS, currently, there is no available treatment approved for RIS. Therefore, our idea of “early treatment administration” might be already late in some cases. In order to detect the individuals who will progress to MS, we need accurate biomarkers. In this review, we present notable research results regarding the underlying pathology of MS, as well as several potentially useful laboratory and neuroimaging biomarkers for the identification of high-risk individuals with RIS for developing MS. This review aims to raise clinicians’ awareness regarding “subclinical” MS, enrich their understanding of MS pathology, and familiarize them with several potential biomarkers that are currently under investigation and might be used in clinical practice in the future for the identification of individuals with RIS at high risk for conversion to definite MS.

## 1. Introduction

Since 1868, when Jean-Marie Charcot first described multiple sclerosis (MS), continuous research in the field of neuroimmunology, particularly in MS, as well as the implementation of advanced laboratory and neuroimaging methods has led to the development and optimization of the diagnostic criteria for MS [1,2]. The diagnostic criteria, with the incorporation of paraclinical tests, aim to establish not only accurate but also early MS diagnoses.

So far, numerous clinical studies have revealed the value of early treatment administration, which is associated with slower disease progression and improved late outcomes, regarding both disability accumulation and cognitive impairment [3,4,5]. For this reason, the use of several disease-modifying therapies (DMTs) has already been approved in individuals with clinically isolated syndrome (CIS), before the progression to definite MS [6].

Although, according to studies, half of the individuals with radiologically isolated syndrome (RIS) will convert to MS within 10 years with a significant proportion progressing to primary progressive MS (PPMS), currently, there is no available treatment for RIS [7]. However, research based on histopathological findings from experimental animals and postmortem tissue, as well as neuroimaging results, suggests the presence of underlying neuroinflammation in asymptomatic individuals, even in the normal-appearing white matter (NAWM) in the absence of lesions on magnetic resonance imaging (MRI) [8,9,10,11]. Therefore, despite our effort to provide early treatment to individuals with MS, the aforementioned findings suggest that our understanding of “early” administration might already be “late” for these individuals who will progress to definite MS.

The identification and validation of accurate laboratory and neuroimaging biomarkers are crucial for the detection of individuals with RIS at high risk for conversion to definite MS. Currently, several laboratory biomarkers, found both in the serum and cerebrospinal fluid (CSF), as well as neuroimaging biomarkers, are under investigation. Furthermore, the identification of biomarkers may provide valuable insights into the pathology of the disease and might reveal new targets for the development of novel therapeutic agents.

## 2. Methods

The present article reviews the current literature regarding novel research results on laboratory and radiological biomarkers of MS that may have a predictive value for the identification of individuals with preclinical MS who will progress to definite MS. For the conduct of this review, the databases Medline and Scopus were accessed. We used the terms “RIS”, “subclinical MS”, “CIS”, “MS prodrome”, “laboratory biomarkers in MS”, and “neuroimaging biomarkers in MS” in order to retrieve all the related articles published in the last decade from the literature. Finally, we assessed and combined all the valuable knowledge we scraped together, which is presented in this current review.

## 3. Evolution of MS Criteria

In the time of precision medicine, the development of diagnostic criteria acts as a cornerstone in the establishment of early and accurate diagnoses of various medical disorders. Diagnostic criteria for MS have largely evolved through time. Initially, only clinical information was used whereas later, paraclinical tests were included, speeding up the time to diagnosis. Defining the different types and disease courses of MS, elucidating the terms of dissemination in space and time, and determining the roles of neuroimaging and laboratory tests are the key aspects of MS diagnostic criteria.

The clinical triad of nystagmus, intention tremor, and scanning speech was the first description of MS by Jean-Martin Charcot in 1868 [1]. Allison and Millar, in 1954, were the first to form criteria, according to which they defined four distinct types of MS based on clinical signs and features: early disseminated sclerosis, probable disseminated sclerosis, possible disseminated sclerosis, and discarded cases. Additionally, they recognized and separated the disease courses into two distinct groups: probable and possible cases [12].

Schumacher, in 1965, was the first to define “relapse” as the number of new or aggravated symptoms lasting for at least 24 h with an associated objective change on a clinical examination and preceded by at least a month of stability or improvement. He also acknowledged progression as a disease course, and he mentioned the development of a quantitative method of neurological function evaluation. Furthermore, Schumacher laid the foundations of the concept of dissemination in time (DIT) and space (DIS). Although McAlpine had already highlighted the usefulness of a detailed medical history in the detection of prior clinical episodes that can be attributed to distinct central nervous system (CNS) events, the Schumacher criteria clearly required either two or more defined relapses, or stepwise progression of signs and symptoms over a period of at least 6 months. Consequently, exacerbations of any other etiology, as well as other non-recurrent neurological diseases were excluded [13]. According to the Schumacher criteria, the involvement of at least two distinct neuroanatomic CNS regions was necessary for the establishment of the diagnosis, whereas laboratory tests were only used for the exclusion of other diseases [14].

A decade later, in 1977, McDonald and Halliday built upon the Schumacher criteria and defined five disease types: clinically definite, early probable or latent, progressive probable, progressive possible, and suspected. For the diagnosis of definite MS, the fulfillment of six criteria was required: (1) the presence of objective neurological abnormalities, (2) involvement of two or more distinct anatomical regions of the CNS based on the medical history or clinical examination, (3) objective evidence that the ongoing process predominately reflects white matter (WM) involvement, (4) evolution over time, (5) disease onset between 10–50 years old, and (6) exclusion of other diagnoses [15].

In 1983, the Poser criteria, which also shared the core principles of the Schumacher criteria, divided MS into two main types: definite and probable MS. The terms definite and probable MS refer to two separate attacks with evidence of either two or one anatomic site typical of MS, respectively. The innovation of these criteria was the incorporation of laboratory tests as part of the diagnostic process, which marked the end of a purely clinical diagnosis of MS. In particular, the presence of oligoclonal immunoglobulin G (IgG) bands in the CSF but not in the serum, or an increased IgG index in CSF were equal to the presence of a second lesion for the demonstration of DIS [16].

Finally, the McDonald criteria and their revisions created a new era in the field of MS diagnosis. The McDonald criteria focused on facilitating their implementation both in medical research and clinical practice. The integration of paraclinical tests including MRI, visual evoked potentials (VEPs), and CSF markers; the definition of primary progressive MS (PPMS); and the classification of diagnostic categories into three (MS, possible MS, and not MS), as well as the gradual simplification of the criteria through the McDonald revisions over time, contributed to earlier and more accurate diagnoses and paved the path for the consequent recognition of an MS prodrome. The 2001 McDonald criteria were the first to include MRI findings in the demonstration of both dissemination in space and time [17]. Through the revisions, time restrictions in MRI scanning were finally withdrawn; initially, in the 2005 McDonald criteria, a new lesion on MRI 30 days after baseline could fulfill DIT, whereas, in the 2010 and 2017 McDonald criteria, the presence of simultaneous gadolinium-enhancing (Gd+) and non-enhancing lesions represented DIT [18]. Regarding DIS, the 2001 McDonald criteria adopted the Barkhof and Tintore criteria [19], and in the 2005 revision, the role of a spinal cord lesion was further elucidated [18]. However, later, in the 2010 and 2017 revisions, the criteria were simplified as to the number of lesions and the role of Gd-enhancing lesions [2,20]. Moreover, the 2017 McDonald criteria incorporated cortical lesions, even though current MRI techniques have a limited ability to identify such lesions. However, they did not integrate the optic nerve as a fifth distinct CNS location (in addition to periventricular, juxtacortical, infratentorial, and spinal cord) nor adopted the number of three periventricular lesions according to the European Magnetic Resonance Imaging in Multiple Sclerosis (MAGNIMS) network recommendations [21]. CSF markers, namely oligoclonal bands (OCBs) and the IgG index, were initially used as evidence of DIS in the 2001 McDonald criteria; however, in the 2017 McDonald criteria, they constitute an alternative for the demonstration of DIT. A retrospective study of patients with clinically isolated syndrome (CIS) with DIS on MRI showed increased specificity of OCBs in CSF for the diagnosis of MS and supported their use in DIT. In 2013, disease activity, defined as a clinical relapse and/or new or enlarging or Gd-enhancing (Gd+) lesion on MRI, and disease progression were redefined as two different, but possibly simultaneously present entities. Finally, it was in the 2005 McDonald criteria when aquaporin-4 (AQP4) serum antibody testing for neuromyelitis optica spectrum disorder (NMOSD) was first recommended to be performed in all cases with clinical manifestations suggestive of NMOSD [18]. The McDonald criteria were initially formed based on Caucasian European—North American populations and their application to other populations, such as children under the age of 12 years old and adults with late-onset MS, particularly those above 50 years old, is challenging, despite the fact that they are considered applicable to these age groups [2]. Through the evolution of MS diagnostic criteria, the gradual integration of neuroimaging and laboratory biomarkers has clearly contributed not only to a more precise diagnosis but also to a reduction in the time to definite diagnosis since the onset of the disease [22].

The ultimate goal of the optimization of the diagnostic criteria is to both ensure an early and accurate MS diagnosis and decrease the probability of misdiagnosis. Two distinct neurological entities that do not fulfill the criteria for definite MS have been recognized: CIS and RIS.

## 4. Clinically Isolated Syndrome

Clinically isolated syndrome (CIS) is defined as a sole clinical episode with symptoms and signs suggestive of either a monofocal or multifocal inflammatory demyelinating CNS event that lasts at least 24 h, with or without remission, in the absence of fever or infection. It is similar to a typical MS relapse, occurring for the first time in an individual without a diagnosis of MS. Therefore, the criteria for dissemination either in space or time, or both, are not fulfilled [2]. Typical manifestations of CIS include involvement of the optic nerve, brainstem, or spinal cord. Additionally, supratentorial or cerebellar syndromes can also occur [23]. According to several studies, the overall risk of conversion to MS in individuals with CIS and MRI lesions suggestive of MS varies from 60% to 80%. The strongest predictors of this conversion appear to be the number of MRI abnormalities characteristic of MS and the existence of OCBs in CSF [24]. However, by evaluating the presence of OCBs in CSF as an alternative to demonstrate DIT in the 2017 revision of the McDonald criteria, the cases of CIS have been substantially reduced since people with a sole clinical episode typical of MS, MRI lesions fulfilling DIT, and positive OCBs in CSF can now have a definite diagnosis of MS [25]. Moreover, a single-center prospective study also suggests a high predictive value of subclinical multimodal evoked potential abnormalities [26]. Early treatment of MS has become more and more popular in clinical practice, and thus the initiation of DMTs in individuals with CIS has already been established. In the United States (US), most of the DMTs approved for the relapsing forms of MS are also approved for patients with CIS and MRI lesions typical of MS in the brain or spinal cord [6]. In Europe, the European Academy of Neurology (EAN) and the European Committee for Treatment and Research in Multiple Sclerosis (ECTRIMS) recommend the initiation of glatiramer acetate or interferons for typical CIS [27].

## 5. Radiologically Isolated Syndrome and Subclinical MS

Radiologically Isolated Syndrome (RIS) was first introduced in 2009 with the establishment of the Okuda criteria [28]. The Okuda criteria are essentially focused on the demonstration of DIS on CNS MRIs by incorporating the pre-existent Barkhof criteria [19]. According to the Okuda criteria, the incidental identification of at least one T2-hypointense lesion on CNS MRI involving at least two of the following CNS locations: spinal cord, infratentorial, cortico-juxtacortical, and periventricular white matter, in the absence of neurological signs and symptoms correspondent with MS and after the exclusion of any other diagnoses potentially responsible for the neuroimaging abnormalities, particularly vascular-related abnormalities, is necessary for the establishment of RIS [28]. Following the MAGNIMS consensus recommendations, the definitions of DIS and DIT, as they had been stated in the revised McDonald criteria, were incorporated into the Okuda criteria [29]. Furthermore, the MAGNIMS consensus highlighted the importance of the absence of any clinical signs and symptoms compatible with MS, which must be confirmed by a meticulous medical history and clinical examination [21,26]. Therefore, great caution is required when individuals with CNS MRIs suggestive of demyelination, typical of MS, present with symptoms such as chronic headache, migraine, seizures, psychiatric disturbances, and cognitive impairment, as well as paroxysmal symptoms, since it could be subclinical MS [30].

Although RIS refers to asymptomatic individuals with MS-like lesions found either on their brain or spinal cord MRIs incidentally, a significant proportion converts to MS in the following years. Notably, a large multinational study including individuals with RIS revealed that about one-third of individuals with RIS convert to MS within 5 years from onset, whereas more than half of them convert to definite MS within 10 years, with almost 12% particularly converting to PPMS [7]. So far, several studies have been conducted to unveil the prognostic factors associated with the conversion from RIS to MS. Until now, male sex and a young age at onset have been identified [31]. The presence of infratentorial and/or spinal cord lesions at baseline and/or gadolinium-enhancing (Gd+) lesions either at baseline or at follow-up on MRI are suggestive of subclinical MS and they are strongly associated with an increased risk of conversion as well [32]. Additional findings, such as the presence of OCBs in CSF, cognitive impairment, a high T2 hyperintensity lesion load, DIT on MRI, and optic nerve demyelination revealed by visual evoked potentials (VEP), are also suggestive of subclinical MS [33].

Regarding treatment administration, studies of individuals with RIS have shown controversial results, suggesting that treatment is beneficial for those with a high risk of conversion from RIS to MS [34]. In particular, two studies examining the efficacy of teriflunomide and dimethyl fumarate in RIS, respectively, revealed promising results with regard to the time to clinical symptom onset [35,36]. Furthermore, according to a study performed in the US, in which 239 MS specialists participated, 79% of them declared that they would not prescribe DMTs to an individual with RIS, but all of them would obtain a follow-up MRI instead, with 32% doing so within a year, whereas 94% would additionally obtain a spinal cord MRI as part of the evaluation. However, 80% of MS specialists agreed that they would proceed to treatment administration for an individual with more than two gadolinium-enhancing (Gd+) lesions [37]. Currently, treatment administration has not been approved for individuals with RIS. However, due to the promising results for treatment administration in high-risk individuals with RIS, it is very possible that treatment administration for RIS will be approved in the future. The presence of high-risk individuals in stages as early as RIS, as well as our need to identify them in this preclinical stage, has led to the development of the concept of MS prodrome.

## 6. MS Prodrome

The term prodrome, which is used in innumerable infectious and inflammatory diseases, refers to the group of signs, symptoms, as well as other abnormal findings that precede the occurrence of characteristic features of a disease. The onset of the prodromal phase may begin many years, even a decade, earlier. Additionally, not all individuals have to present with the same clinical picture, and therefore symptoms may greatly vary. The prodrome of a disease takes place during the latent period, which corresponds to the time between the onset of the underlying pathophysiological mechanism of the disease and the occurrence of the first typical features.

According to population-based studies examining the 5-year time prior to the onset of MS, an increase in healthcare usage by individuals who developed MS a few years later has been observed, including hospitalizations and physician visits, particularly to psychiatrists, prescriptions, as well as contraceptives by females. These observations suggest a behavioral change that could also be a feature of MS prodrome. Furthermore, according to these studies, the most frequently mentioned complaints are sleep disturbances, bladder dysfunction, bowel disturbances, fatigue, depression, anxiety, pain, migraine/headache, cognitive impairment, and anemia. All these features could be manifestations of the MS prodrome [38].

Although RIS is not characterized as a distinct MS subtype, ground-breaking revelations pose important questions regarding our understanding of MS pathology and pave the way towards the concept of the MS prodrome. Histopathological findings of NAWM in postmortem tissue revealed significant glial activation around early active lesions, particularly involving hypertrophic reactive astrocytes, as well as activated M1 microglia [9]. Therefore, despite the absence of typical MS symptoms, the insidious pathobiology of the disease may be silently working behind one sole lesion. Additionally, neuroimaging findings with MRI and volumetric analyses revealed significant changes in the NAWM of individuals with MS. These changes, reflecting volume loss on MRI despite the absence of lesions, are attributed to neuroaxonal damage, demyelination, and gliosis [11]. Additionally, experiments in animal models, particularly in experimental autoimmune encephalomyelitis (EAE) rats, revealed the presence of reactive astrocytes in the preclinical, asymptomatic phase of the disease [8].

## 7. Early Treatment Administration

Nowadays, neurologists and MS specialists have a wide range of available DMTs in their armory. Numerous studies examining the appropriate treatment strategy for people with MS (PwMS) have been conducted. Currently, the available DMTs are divided into medium- and high-efficacy drugs, and therefore two treatment strategies result from this categorization: early intensive treatment (EIT) starting with high-efficacy DMTs and escalation treatment, starting with medium-efficacy DMTs and switching to high-efficacy DMTs if needed, according to disease activity [39].

A large cohort study, recruiting individuals with RRMS, revealed that frequent relapses and shorter interattack intervals within the first 2 years from MS onset are associated with more rapid deterioration and more severe disability, whereas the frequency of relapses beyond the first two years does not seem to be related to late outcomes [40]. Another study identified male gender, young age at onset, residual deficit after the first relapse, and the frequency of relapses within the first two years as independent predictive factors of disability progression. These results demonstrate a two-stage process of disability progression in RRMS: the first stage seems to be associated with the extent of focal neuroinflammation and the entry of immune cells into the CNS, and the second stage that is independent of focal neuroinflammation and it is mostly associated with neurodegeneration, driven mainly by reactive astroglia and microglia [41]. Based on this theory, an early intensive treatment strategy with HETs would be more appropriate for these patients. Several studies researching the efficacy of HETs on disease progression and disability accumulation revealed that patients treated with HETs had a significantly lower risk of conversion to SPMS [42,43]. Additionally, early treatment administration decreased the risk of conversion to SPMS, whereas delayed treatment administration increased the risk of progression to SPMS and the probability of reaching higher EDSS scores in a shorter time [44]. Considering these results, the 2018 American Academy of Neurology (AAN) recommendations regarding the choice of treatment strategy for MS stated that HETs should be prescribed for highly active RRMS individuals [45]. Consequently, the window opportunity is early in the course of RRMS, and therefore, early treatment administration is needed to slow disability progression.

Although several factors have been proposed as predictors of disability in MS patients, including demographic characteristics, clinical, and radiological findings, their predictive value in disability progression in individuals with RIS after their conversion to definite MS has not been examined yet. Demographic characteristics such as the male sex, obesity, smoking status, low vitamin D levels, motor or cerebellar signs at onset, a short interattack period between the first and the second relapse, early cognitive impairment, a high relapse rate in the first years of the disease, spinal cord lesions, high lesion load, and brain atrophy have all been associated with increased disability in MS patients [46,47,48,49]. Moreover, the use of specific serum biomarkers, such as neurofilament light chains (NfLs) and glial fibrillary acidic protein (GFAP), as disability predictors is also under investigation [50,51]. The predictive value of these demographic and radiological characteristics in disability prediction in RIS patients after conversion to definite MS needs to be assessed.

Apart from DMT administration, countless studies have unveiled the favorable effects of physiotherapy on individuals with MS, including physical, cognitive, and occupational therapy interventions, as well as neurostimulation techniques, such as transcranial direct-current stimulation (tDCS) and repetitive transcranial magnetic stimulation (rTMS) [52,53,54,55,56,57,58]. Yet today, the effects of these techniques on individuals with RIS who will convert to definite MS with regard to disability progression have not been studied.

On one hand, since RIS seems to be the onset of MS in a significant proportion of individuals with MS, neuroinflammation accompanied by glial activation has been observed around early active lesions, and half of the individuals with RIS progress to MS within 10 years, with almost 12% converting to PPMS; treatment administration seems to be already delayed [7]. On the other hand, despite the beneficial effects of DMTs on disease activity and progression, these medications are associated with serious side effects, such as an increased risk of infections that could be potentially life-threatening in some cases, and therefore they cannot be prescribed prophylactically to all individuals with RIS [59]. Moreover, prophylactic administration of DMTs would also be cost-effective. Consequently, precise identification of the individuals with RIS who will progress to MS is necessary. In order to be able to identify these individuals early, the development of accurate biomarkers is absolutely crucial. Furthermore, the development of biomarkers will enrich our knowledge and understanding of MS pathology, and they will also provide potential targets for the development of novel therapies [60].

## 8. MS Pathology and Biomarkers

### 8.1. MS Pathology

The evolution of research methods with the development of innovative laboratory techniques, as well as the use of novel therapeutic modalities in PwMS provided valuable insights into MS pathology. Despite the tremendous effort of scientists to understand the exact underlying pathophysiological mechanism of the disease and develop curative treatments for all PwMS, currently, the order of events taking place in MS pathology is still a matter of debate and there is no available treatment halting disease progression overall.

Although the administration of HETs decreased the number of relapses and provided better control over neuroinflammation, disease progression manifesting as disability accumulation and cognitive impairment remains a great burden both for PwMS and their families. These findings highlighted the significance of neurodegeneration in MS progression and, consequently, the key role of glia, particularly astrocytes and microglia, in disease progression [61]. In order to develop accurate laboratory biomarkers of neuroinflammation and neurodegeneration, the cells involved in the disease process, as well as the complex interconnections between them, need to be identified [62].

Notwithstanding the current debate on the sequence of events taking place at the onset of MS pathology, neuroscientists believed for decades that neuroinflammation starts with the crossing of the blood–brain barrier (BBB) by CD4+ T helper (Th) cells and B cells that enter the CNS due to a disruption in the BBB and recognize myelin-specific antigens, which they present to Th cells and trigger an inflammatory response [63,64]. The inflammatory response initiates with the release of pro-inflammatory cytokines and chemokines, which lead to the recruitment of resident microglia, as well as macrophages, CD8+ cells, and astrocytes. The accumulation of these cells leads to the formation of an active lesion [64,65].

In EAE models, myelin destruction and accumulation in the extracellular space (ECS) exceeds the phagocytic capacity of microglia, which are normally responsible for the removal of debris via phagocytosis, resulting in myelin phagocytosis from astrocytes too. “Myelin-rich” astrocytes become hypertrophic and surround the active lesion [66]. Although astrocytes, which are normally responsible for the stimulation of oligodendrocyte progenitor cells (OPCs) that differentiate into mature oligodendrocytes, induce OPC differentiation, these cells are not fully capable of migrating into active lesion areas, and therefore active lesions remain partially regenerated until remission, when oligodendrocyte regeneration overcomes myelin destruction [62,63]. Later, once-active lesions become scar tissue over time [66].

On the other hand, in progressive MS, extracellular myelin deposition is more extensive and remyelination is dysfunctional, which leads to the existence of chronically active lesions [66]. In progressive MS, the main underlying mechanism responsible for the clinical outcome of the disease is neurodegeneration, triggered by smoldering neuroinflammation over the years [67]. Myelin deposition and consequent phagocytosis by astrocytes turn them into reactive, hypertrophic astrocytes and microglia into M1 activated microglia [68,69]. Both cell types release various cytokines and chemokines after myelin phagocytosis as a response to neuroinflammation, triggering further cellular recruitment and neurodegeneration [70].

Several substances released from all the aforementioned cell types that could be detected and quantified and are found either in the serum or CSF would be potentially useful laboratory biomarkers for the identification of individuals with RIS who will develop MS and would benefit from early treatment administration.

### 8.2. Biomarkers

#### 8.2.1. Laboratory Biomarkers

Although the sequence of events in the pathology of the disease is not definite, adaptive immune cells from the periphery cross the BBB and enter the CNS [64]. OCBs are mainly IgGs produced by clonally expanded B cells intrathecally [71]. OCBs were the first validated biomarkers and have been used as an accurate biomarker in the diagnosis of MS for decades, as well as for the conversion of CIS to MS [72]. According to long-term studies of individuals with RIS, the presence of OCBs in CSF has prognostic value, predicting the conversion from RIS to MS [73]. Additionally, the identification of OCBs in the CSF of individuals with CIS and RRMS has been associated with higher EDSS scores and a shorter time to severe disability [33]. Therefore, the detection of OCBs in the CSF of individuals with RIS correlates both with clinical conversion and disease activity. However, according to study results, no correlation between the conversion time and OCB detection in the CSF was seen in individuals with RIS. Therefore, the predictive value of OCBs in RIS patients is higher for disease activity and not clinical conversion [73].

Other potential biomarkers related to B cells are kappa free light chains (kFLC), intrathecal IgM, and distinct B cell profiles. The measurement of kFLC, which is also used for the estimation of intrathecal Ig production, seems to be more precise than OCBs and the IgG index in CSF in the conversion from CIS to MS [74,75]. So far, interestingly, studies of patients with RIS show mixed results [76,77]. Therefore, larger studies need to be conducted for the assessment of kFLC as a biomarker of RIS. Apart from OCBs and IgGs, the identification of intrathecal IgM production correlates with higher disease activity and has a prognostic value both for RRMS and PPMS [78]. However, the predictive capacity of CSF IgM in RIS has not been assessed yet. Finally, a study examining the different profiles of B cells in people with CIS revealed that a specific B cell subtype, particularly double-negative IgD2/CD272 B cells, increased in these individuals [79]. Similar studies of individuals with RIS may reveal valuable results and provide new biomarkers.

Neurofilaments are intermediate filaments found in the axons and neurons, located both in the periphery and the CNS. Any cause of axonal damage, either due to direct injury or neurodegeneration, leads to the release of neurofilament degradation products in the ECS. NfLs have been traced both in the CSF and the serum of PwMS after entering the systemic circulation via the lymphatic system. Until recently, accurate measurements of NfL levels were only feasible in CSF [80]. According to studies, accurate measurements of NfL levels in CSF correlate with the presence of active disease in patients with CIS [81]. A Swedish study examining the potential use of NFLs and OCBs in CSF as biomarkers in patients with RIS revealed that high NFL levels in the CSF of RIS patients correlated with an increased risk of conversion to CIS or RRMS. However, the 2010 McDonald criteria were used for the definition of CIS [81]. Another study of RIS patients showed that although definite MS, including individuals with both PPMS and RRMS, can be distinguished from RIS with the use of NfL levels in CSF, CSF levels were similar for RIS, CIS, and early MS patients [82]. On the other hand, recent advances in technology and their implementation in laboratory methods permitted the accurate measurement of NfL levels in serum. According to a hypothesis, since NfLs enter the systemic circulation via the lymphatic system, the amount of NfL levels in serum possibly fluctuates according to the degree of BBB disruption and therefore disease activity [80]. So far, serum NfL level measurements correlate with NfL levels in CSF [83,84]. Moreover, studies on serum NfL levels in individuals with CIS and MS showed that not only serum NfL levels are associated with the activity of the disease, patient response to DMT, and the long-term outcomes but also that serum NfL levels represent an independent factor of relapse in CIS and early MS individuals [85,86,87,88]. Despite serum NfL levels being significantly higher in people who were diagnosed with MS within the next 6 years, in a large epidemiological study performed in the US Army, serum NfL levels have not been studied in RIS patients yet [89]. Further investigations are needed to assess the potential use of serum NfL levels as biomarkers in RIS patients.

GFAP is a major intermediate filament protein found in astrocytes. GFAP levels increase proportionally with the extent of astrocytic activation [90]. Since astrocytes play a key role both in active disease but also in neurodegeneration and disease progression, the assessment of their prognostic value is of great importance. Results from studies on CSF GFAP levels revealed a correlation between GFAP levels in CSF and the various MS phenotypes [91]. On the other hand, serum GFAP levels correlate with an older age, higher Expanded Disability Status Scale (EDSS) score, longer disease duration, and progressive course [92,93]. According to these results, GFAP may be a strong biomarker for disease severity and potentially the conversion to progressive disease. Consequently, the investigation of GFAP levels in individuals with RIS converting to PPMS might provide useful results.

Chitinase 3-like protein 1 (CHI3L1), also referred to as YKL-40, is another protein released by activated astrocytes, microglia, and macrophages in the setting of inflammation, either acute or chronic [94,95]. Until now, all studies examining the prognostic value of CHI3L1 in the CSF of individuals with RIS for the conversion to MS came out negative [68,96,97]. However, studies of individuals with CIS revealed that CSF CHI3L1 levels can predict the CIS conversion to definite MS and disability progression [98]. Additionally, serum CHI3L1 levels seemed to predict the CIS conversion to RRMS, and they were also higher in people with progressive disease compared to those with RRMS, confirming its implication with neurodegeneration and disease severity [99,100]. Serum CHI3L1 levels in individuals with RIS have not been studied yet. Furthermore, apart from the investigation of CHI3L1 levels in serum, the presence of two other molecules, chitotriosidase and chitinase 3-like protein 2 (CHI3L2), in the CSF of RIS patients can be studied [101,102].

**Figure 1:**
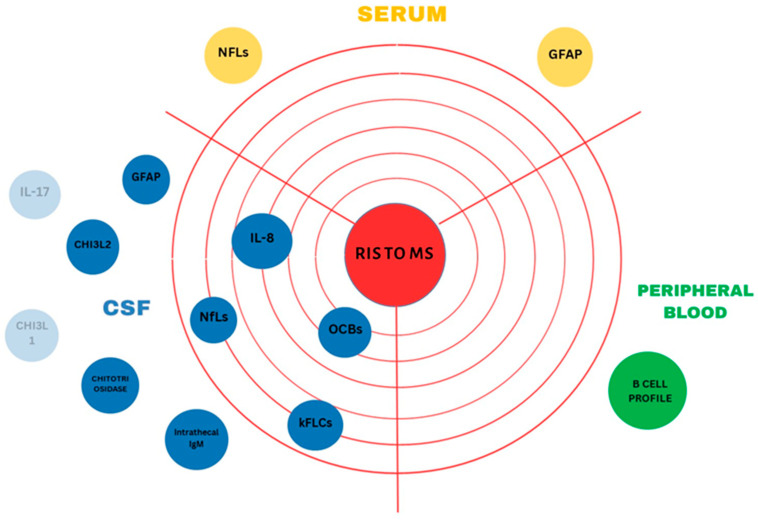
The potentially predictive laboratory biomarkers for the progression of radiologically isolated syndrome (RIS) to definite multiple sclerosis (MS) are summarized. Laboratory biomarkers in blue are found in the cerebrospinal fluid (CSF), whereas those in yellow are found in the serum, and the one in green is retrieved from peripheral blood. Concentric circles are used to visualize the predictive value of each biomarker according to the currently available research results. Biomarkers closer to the center have been found to be more strongly associated with RIS progression to definite MS compared to those located in the outer layers of the circle. Biomarkers located outside the circle have not been assessed yet for their predictive value in RIS conversion to MS. Transparent biomarkers have already been assessed and there was no association found.

Interleukin-8 (IL-8) is a proinflammatory chemokine released by astrocytes and microglia in response to active inflammation, inducing further cellular recruitment [103]. Studies on IL-8 levels in CSF revealed that high CSF IL-8 levels can predict clinical conversion both from CIS to definite MS and, most importantly, from RIS to MS [104]. However, the study included only 18 individuals with RIS, and therefore the conduct of larger-scale studies is necessary for proper assessment. In contrast to IL-8, studies of both serum and CSF IL-17A levels produced negative results regarding its predictive value as a biomarker in individuals with RIS, even though experiments on EAE models had previously outlined its role as an important pro-inflammatory chemokine [105,106]. Figure 1 shows the potentially available laboratory biomarkers that could predict RIS conversion to definite MS, according to current research results.

Figure 1 Potentially predictive laboratory biomarkers for RIS progression to definite MS.

#### 8.2.2. Neuroimaging Biomarkers

Apart from laboratory biomarkers, the identification of neuroimaging biomarkers in MRI is crucial in order to identify which findings in the MRIs of individuals with RIS are highly suggestive of future conversion to definite MS. Until now, the presence of infratentorial and spinal cord lesions at baseline, as well as the presence of gadolinium-enhancing (Gd+) lesions either at baseline or at follow-up, have been confirmed as biomarkers with prognostic value for RIS conversion to definite MS [107]. Recently, the predictive value of three MRI findings in PwMS has drawn significant attention: the central vein sign (CVS), the paramagnetic rim sign (PRS), and the enlargement of choroid plexuses [107,108].

The term central vein sign refers to a vein inside a WM lesion that is visualized as T2 hypointensity in the MRI. CVS findings are seen in all MS phenotypes, including individuals with RIS. The great value of CVS is its increased specificity for MS, making it a potentially suitable biomarker for MS diagnosis [109]. However, CVS has been identified in MRIs of individuals with other diseases, such as diabetes mellitus and systemic lupus erythematosus (SLE), and therefore its sensitivity, as well as its prognostic value, in MS diagnosis are currently under investigation [109,110]. Overall, its role as a valuable biomarker, particularly in RIS, needs to be explored [111].

The term paramagnetic rim sign (PRS), found on chronically active WM lesions, refers to the presence of a central core characterized by demyelination surrounded by a rim of iron-laden immune cells, including macrophages and resident microglia [112]. Histopathological findings from chronic active lesions reveal iron phagocytosis by microglia [113]. Consequently, PRS demonstrates the presence of gliosis around a chronically active lesion, and therefore a smoldering lesion in the setting of disease progression [114]. PRS has a typical appearance on T2-weighted fluid-attenuated inversion recovery (FLAIR) images and it is highly associated with increased disability in MS patients [115]. PRS, together with CVS, is also being examined as a candidate imaging biomarker for MS. Moreover, PRS must be evaluated as a potential imaging biomarker for RIS conversion to definite MS, particularly PPMS, since it is strongly associated with smoldering lesions [116].

Last but not least, MRI studies of PwMS have identified the presence of choroid plexus enlargement in PwMS compared with healthy controls [117,118]. Additionally, choroid plexus enlargement and inflammation had already been observed in the presymptomatic phase in EAE models, suggesting that the choroid plexus could be a potential gateway for CNS lymphocytic infiltration in MS patients [119]. A recent, small-scale retrospective study of individuals with RIS revealed similar results between RIS and definite MS, showing significant choroid plexus enlargement compared with healthy controls. Although these results seem to be promising, according to the follow-up of individuals with RIS and choroid plexus enlargement, there was no differentiation observed between the results of the individuals who progressed to definite MS and those who did not [120]. Therefore, large-scale studies need to be conducted in order to properly assess the prognostic value of choroid plexus enlargement in multiple sclerosis patients. Moreover, choroid plexus enlargement has also been observed in individuals with other disorders, including Alzheimer’s disease, depression, and schizophrenia [121,122,123]. Table 1 summarizes all potential predictive biomarkers that could be used for the recognition of RIS progression to definite MS found either via laboratory or neuroimaging techniques, and the currently available research results.

In conclusion, this article intends to present all the novel biomarkers that may be potentially used concerning the conversion from the preclinical stage of MS to definite MS, according to the available recent research results. The current review highlights the need for further investigations of the predictive value of these biomarkers. Unfortunately, a critical analysis of their predictive value cannot be performed at this moment due to the lack of sufficient number of results from large-scale studies for the vast majority of these biomarkers.

## 9. Conclusions

Early treatment administration is crucial in PwMS in order to slow disease progression and consequently limit both severe disability and cognitive impairment. Despite our efforts to treat individuals with MS as early as possible, our estimation of “early MS” may already be late, since the onset of MS pathology can occur prior to the appearance of clinical manifestations. Since one-third of individuals with RIS will convert to definite MS, their identification with the use of readily available laboratory and neuroimaging biomarkers with high predictive value is necessary. Additionally, a cost effectiveness analysis of these biomarkers needs to be performed in order to decide which biomarkers could be implemented and routinely used in everyday clinical practice. Yet today, neither a specific antibody nor a specific biomarker has been identified for MS. Recent research results revealed the tight associations between Epstein–Barr virus (EBV) infection and MS, and its potential implication in the pathogenesis of the disease, probably via mechanisms of molecular mimicry, particularly in the presence of specific human leukocyte antigens (HLA) alleles [124]. Despite the evolution of laboratory and neuroimaging techniques, further research is needed to yield accurate biomarkers for MS in order to enrich our understanding of disease pathology, change our treatment strategies, and potentially develop novel therapeutic treatments so that we can provide better care for our patients and truly ameliorate their quality of life.

## Figures and Tables

**Table 1:** Potential predictive biomarkers for RIS progression to definite MS.

Biomarker	Examination	Predictive Value for RIS Conversion to MS
OCBs	CSF analysis	Promising results
kFLC	CSF analysis	Mixed results
Intrathecal IgM	CSF analysis	Not assessed yet
B cell profile	Peripheral blood analysis	Not assessed yet
NfL	CSF analysis	Mixed results
	Serum analysis	Not assessed yet
GFAP	CSF analysis	Not assessed yet
	Serum analysis	Not assessed yet
CHI3L1	CSF analysis	Negative results
CHI3L2	CSF analysis	Not assessed yet
Chitotriosidase	CSF analysis	Not assessed yet
IL-8	CSF analysis	Promising results
IL-17	CSF analysis	Negative results
CVS	MRI	Not assessed yet
PRS	MRI	Not assessed yet
CP enlargement	MRI	Mixed results

RIS: radiologically isolated syndrome, MS: multiple sclerosis, OCBs: oligoclonal bands, CSF: cerebrospinal fluid, kFLC: kappa free light chains, IgM: immunoglobulin M, NfL: neurofilament light chains, GFAP: Glial fibrillary acidic protein, CHI3L1: Chitinase 3-like protein 1, CHI3L2: Chitinase 3-like protein 2, IL-8: Interleukin 8, IL-17: Interleukin 17, CVS: central vein sign, PRS: paramagnetic rim sign, CP: choroid plexus.

## Data Availability

Not applicable.
